# ATP releasing channels and the ameliorative effects of high intensity interval training on diabetic heart: a multifaceted analysis

**DOI:** 10.1038/s41598-024-57818-0

**Published:** 2024-03-26

**Authors:** Siyavash Joukar, Mohammad Amin Rajizadeh, Mohammad Abbas Bejeshk, Samaneh Sadat Alavi, Fatemeh Bagheri, Mohammad Rami, Kayvan Khoramipour

**Affiliations:** 1 Department of Physiology and Pharmacology, Afzalipour Medical Faculty, Kerman University of Medical Sciences, Kerman, Iran; 2 Neuroscience Research Center, Institute of Neuropharmacology, Kerman University of Medical Sciences, Kerman, Iran; 3 Cardiovascular Research Center, Institute of Basic and Clinical Physiology Sciences, Kerman University of Medical Sciences, Kerman, Iran; 4 Physiology Research Center, Institute of Neuropharmacology, Kerman University of Medical Sciences, Kerman, Iran; 5 Department of Physiology, Faculty of Medicine, Kashan University of Medical Sciences, Kashan, Iran; 6 Legal Medicine Research Center, Legal Medicine Organization, Kerman, Iran; 7 Pathology and Stem Cell Research Center, Department of Pathology, Afzalipour Medical Faculty, Kerman, Iran; 8 Department of Sport Physiology, Faculty of Sport Sciences, Shahid Chamran University of Ahvaz, Ahvaz, Iran

**Keywords:** Type 2 diabetes, HIIT, ATP-releasing channels, Inflammation, Physiology, Cardiology

## Abstract

Type 2 diabetes (T2D) can cause severe cardiac complications at functional, histologic and molecular levels. These pathological complications could be mediated by ATP-releasing channels such as Panx1 and ATP receptors, in particular P2X7. The aim of our study was to investigate the effect of high-intensity interval training (HIIT) on T2D-induced cardiac complications at the functional, histopathological and molecular levels, with a particular focus on ATP-releasing channels. 48 male Wistar rats at the age of 8 weeks were randomly allocated into four groups: control (Con), Diabetes (T2D), Training (TR), and Diabetes + Training (T2D + TR). T2D was induced by a high-fat diet plus a low dose (35 mg/kg) of STZ administration. Rats in the TR and T2D + TR groups underwent an 8-weeks training program involving intervals ranging from 80 to 100% of their maximum running speed (Vmax), with 4–10 intervals per session. Protein expression of Interleukin 1β (IL1β), Interleukin 10 (IL-10), Pannexin 1 (Panx1), P2X7R (purinergic P2X receptor 7), NLRP1 (NLR Family Pyrin Domain Containing 1), BAX, and Bcl2 were measured in the heart tissue. Additionally, we assessed heart function, histopathological changes, as well as insulin resistance using the homeostasis model assessment of insulin resistance (HOMA-IR). In contrast to the T2D group, HIIT led to increased protein expression of Bcl2 and IL-10 in the heart. It also resulted in improvements in systolic and diastolic blood pressures, heart rate, ± dp/dt (maximum and minimum changes in left ventricular pressure), while reducing protein expression of IL-1β, Panx1, P2X7R, NLRP1, and BAX levels in the heart. Furthermore, left ventricular diastolic pressure (LVDP) was reduced (*P* ≤ 0.05). Moreover, heart lesion scores increased with T2D but decreased with HIIT, along with a reduction in fibrosis percentage (*P* ≤ 0.05). The results of this study suggest that the cardioprotective effects of HIIT on the diabetic heart may be mediated by the modulation of ATP-releasing channels. This modulation may lead to a reduction in inflammation and apoptosis, improve cardiac function, and attenuate cardiac injury and fibrosis.

## Introduction

Persistently high blood glucose levels, known as hyperglycemia, are the main features of diabetes^1^. According to the World Health Organization (WHO), the number of diabetics was 171 million in 2000 and is estimated to reach 366 million by 2030^2^. Diabetes is considered a leading risk factor for cardiomyopathy, neuropathy, nephropathy, and retinopathy and represents a significant economic burden for countries worldwide^3–5^.

Diabetic cardiomyopathy is a myocardial dysfunction unrelated to coronary atherosclerosis or valvular heart disease^6^. It is associated with cardiac impairments such as myocardial fibrosis^7^ and results in both systolic and diastolic dysfunction, ultimately leading to heart failure^8^. The pathogenesis of diabetic cardiomyopathy is multifactorial and includes factors such as impaired myocardial metabolism and mitochondrial function^7,9^, increased oxidative stress^7^, increased expression of inflammatory cytokines and inflammation^7^, fibrosis, increased apoptosis^9^, and microvascular disease^10^.

In diabetes, the body cannot secrete insulin or react to it. The regulation of insulin secretion is a complex process in which the extracellular adenosine triphosphate (ATP) level plays a crucial role. While it was previously believed that only intracellular ATP levels-controlled insulin secretion, more recent study^11^ has shown that extracellular ATP can effectively regulate insulin secretion. Therefore, ATP-releasing channels could be a therapeutic target in diabetic patients. Among these channels, the connexins and pannexins, especially pannexin 1 (Panx-1)^12,13^, play a central role. Panx-1 acts as a membrane channel with high non-selective permeability to ATP, calcium (Ca^2+^), glutamate and certain inflammatory mediators^14^. In particular, it has been observed that the expression of Panx-1 increases under hyperglycemic conditions^15^.

Panx-1 channels normally remain closed, but various factors such as reactive oxygen species (ROS), increased extracellular potassium, nitric oxide and mechanical stimuli can open these channels. The opening of Panx-1 channels makes the cells sensitive to ATP^16^, which leads to increased extracellular ATP levels. This in turn activates purinergic channels (P2X7, P2X4), a process that requires the stable presence of ATP. However, extracellular ATP is rapidly degraded by ectonucleotides^17^. Consequently, ATP release by Panx-1 may provide a mechanism for the local replenishment of ATP required for P2X4 and P2X7 opening and activity^18^. The opening of P2X4 and P2X7 channels leads to the influx of calcium and potassium into cells. An increase in intracellular potassium concentration causes cleavage of the NLRP3-ASC-NEK7-caspase-1 complex, leading to activation of the inflammasome and production of pro-inflammatory cytokines such as immature IL-1β^19,20^. In addition, cleavage of the ASC-NEK7-caspase-1 complex leads to activation of caspase-1, which matures immature IL-1β and exacerbates inflammation. IL-1β can activate the extrinsic apoptosis pathway by initiating the caspase cascade^20^.

On the other hand, Panx1 channels in the membrane of the endoplasmic reticulum led to Ca^2+^ leakage and activation of the internal apoptosis pathway^21^. In cardiac cells, intracellular calcium overload and ROS lead to mitochondria being involved in calcium absorption. This increases mitochondrial membrane permeability and leads to leakage of mitochondrial cytochrome into the cytosol, activating the intrinsic pathway of apoptosis^22^. In contrast to the extrinsic pathway, in which the caspase cascade plays a crucial role, the intrinsic pathway of apoptosis involves pro-apoptotic Bax and anti-apoptotic Bcl2 proteins, and the ratio of these two proteins determines the overall rate of apoptosis^23,24^.

## Material and methods

### Animal care

The study was conducted with the approval of the Animal Welfare and Ethics Committee of Kerman University of Medical Sciences (KUMS) by KUMS institutional guidelines (Ethics Approval Code: IR.KMU.AH.REC.1400.081) and by ARRIVE guidelines. A total of 48 male Wistar rats with an average weight of 200 g and an average age of 8 weeks were purchased from Kerman University of Medical Sciences Animal Farm. These rats were subsequently housed in polycarbonate cages under controlled environmental conditions that included an average temperature of 22 ± 1.4 °C, humidity of 50 ± 4%, and a 12:12 light: dark cycle. All phases of animal housing and sacrificing the animals were performed according to the rules of the Ethics Committee of Kerman University of Medical Sciences. Following their acclimatization to the laboratory environment, the rats were randomly distributed into four groups, each comprising 12 rats: Diabetes training (TR + T2D), Training (TR), Diabetes Control (T2D), and Healthy Control (Con).

### Induction of Type 2 diabetes

The diabetic groups (T2D and T2D + EX) were first fed with a high-fat diet (HFD) for two months, (HFD produced by Zist Fannavaran Royan Imen Co, Isfahan, Iran) (Table 1)^35^. Then the animals were fasted for 12 h and received a single dose of streptozotocin (STZ) (Sigma Aldrich, CAS number:18883-66-4) intraperitoneally (35 mg/kg). Three days after injection, the animals’ fasting blood glucose (FBG) was measured using a glucometer (Accu-Chek). The blood samples were collected from the tail vein. Animals with FBG above 300 mg/dl were considered diabetic^36^. Specifically, HOMA-IR scores were calculated using the following formula: HOMA-IR = [(fasting glucose (mmol/L) × fasting insulin (μU/mL))/22.5].

**Table 1:** Regular and high-fat diet ingredients.

Diet ingredients	Fat (%)	Carbohydrate (%)	Protein (%)	Fiber (g)	Minerals (g)	Vitamins (g)
Regular diet	10	70	20	50	50	3
High-fat diet	60	20	20	50	50	3

### Training protocol

We used K1 protocol which has been designed in our lab and the details can be fined in our previous publications^35–38^. In summary, the animals in the TR and T2D + TR groups were familiarized with treadmill (made by Tajhiz Gostar Co , Iran) twice a day for 5 days, 10 min per day with a zero-incline and a speed of 8 m per minute^39^. Then they performed an incremental running test to determine their maximum running speed (V_max_). In this test, the rats ran for 2 min at a speed of 6 m per minute and zero -incline, and every 2 min, 2 m per minute were added to the speed until they could not maintain the speed. The last attempt of each rat was considered Vmax^39,40^. The V_max_ was used for designed the HIIT program for eight weeks (80–100% V_max_, 4–10 intervals). The training was initiated after diabetes induction and confirmation (Table 2). At the beginning and end of each session, the rats run on a treadmill for 5 min at an intensity of 40–50% of V_max_ as warm up and cool down, respectively.

**Table 2:** HIIT details.

Week	Slope	Frequency	Intervals	High-intensity interval duration (min)	Low-intensity interval duration (min)	High-intensity interval velocity (%Vmax)	Low-intensity interval velocity (%Vmax)	Total exercise time in a session (min)
1	0	5	4	2	1	80	50	12
2	0	5	4	2	1	85	50	12
3	0	5	6	2	1	85	50	18
4	0	5	6	2	1	90	50	18
5	0	5	8	2	1	90	50	24
6	0	5	8	2	1	95	50	24
7	0	5	10	2	1	95	50	30
8	0	5	10	2	1	100	50	30

### Measurement of physiological heart parameters and blood pressure

Seven animals in each group were used for analyzing heart function. Twenty-four hours after the last training session, animals were weighed and then anesthetized with sodium thiopental (50 mg/kg). A cannula was inserted into the left carotid artery to record blood pressure and the other cannula was inserted into the left ventricle via the right carotid artery. This allowed precise recording of ventricular pressure via a pressure transducer and the PowerLab system^41^. The animals’ trachea was cannulated so that they could be connected to a ventilator if necessary. Systolic arterial pressure (SAP), diastolic arterial pressure (DAP), left ventricular end-diastolic pressure (LVEDP), the maximum positive change in left ventricular pressure (+ dP/dt max, an index of contractility) and the maximum rate of reduction in left ventricular pressure (− dP/dt max, an index of relaxation velocity) were measured and calculated^42^. In addition, it has been shown that the administration of sodium thiopental did not alter the glucose levels of plasma^43–45^.

### Homogenization and measurement of cytokines

Forty-eight hours after the last training session, the animals were euthanized by intraperitoneal injection of a high dose of ketamine (100 mg/kg) and xylazine (80 mg/kg) and blood and heart samples were collected. Left ventricular tissue was first washed with saline and then homogenized in tris buffer (Sigma-Aldrich, Germany) and phenylmethylsulfonyl fluoride (PMSF) using an ultrasonic homogenizer. 0.5 mM PMSF solution was used as a protease inhibitor^46^. The homogenized solution was centrifuged at 5000 rpm for 5 min. The supernatant was taken, and then IL1β (Karmania pars gene Co, Kerman, Iran, CN: KPG-RIL1β Ka, Sensitivity: 2 pg/ml) and IL10 (Karmania pars gene Co, Kerman, Iran, CN: KPG-RIL10 Ka, Sensitivity: 2 pg/ml) levels were measured by a special kit according to the manufacturer's protocol using an ELISA reader^47,48^. All steps were performed at 4 degrees centigrade to prevent the destruction of enzymes and proteins.

### Western blotting method

The Western blotting technique was employed to evaluate protein levels of Panx-1 (SANTA CRUZ, sc-515941), NLRP-1 (abcam, ab91413), P2X7R (SANTA CRUZ, sc-514962), Bax (SANTA CRUZ, sc-7480), and Bcl2 (SANTA CRUZ, sc-492) within the heart. The total protein concentration present in the left ventricle samples was quantified via the Bradford method. For the immune detection step, a Chemi Doc XRS + imaging system (Bio-Rad Company, USA) was utilized. Subsequently, the resulting images were subjected to meticulous analysis employing ImageJ software^49,50^. Notably, β-Actin (SANTA CRUZ, sc-47778) served as the designated housekeeping protein^51,52^. We provided one image in result session for each protein which is a representative of seven protein extracts.

### Histological examination of the heart

The harvested hearts were promptly immersed in 10% formalin for preservation. Subsequently, a comprehensive evaluation was conducted utilizing hematoxylin/eosin (H&E) and Masson trichrome staining. A variety of pathological indices within the heart were assessed, encompassing inflammation, lesions, and necrosis (by a blinded pathologist). When interpreting H&E stains, changes were systematically graded according to the specific criteria as outlined in Table 3. Additionally, the percentage of fibrotic changes were reported as a percentage^42^.

**Table 3:** Histological score system.

Score	Index
	Pathologic changes
0	unchanged or negative
1	minimal (local myocyte lesions)
2	Mild (multifocal destruction with mild degrees of inflammatory processes)
3	Moderate (widespread degradation of myofibrils or diffuse inflammatory processes)
4	severe (marked) (necrosis + diffuse inflammatory processes)

### Statistical analysis

Data analysis was accomplished utilizing Graph pad Prism software version 8. The Shapiro test determined the normal distribution of data. While repeated measure ANOVA were used for analyzing FBG data, other analysis was performed by One-way ANOVA. Tukey post hoc test was utilized to find significant differences between groups in all analysis. The significance level was considered less than 0.05.

## Results

### Blood glucose & HOMA-IR

**Figure 1:**
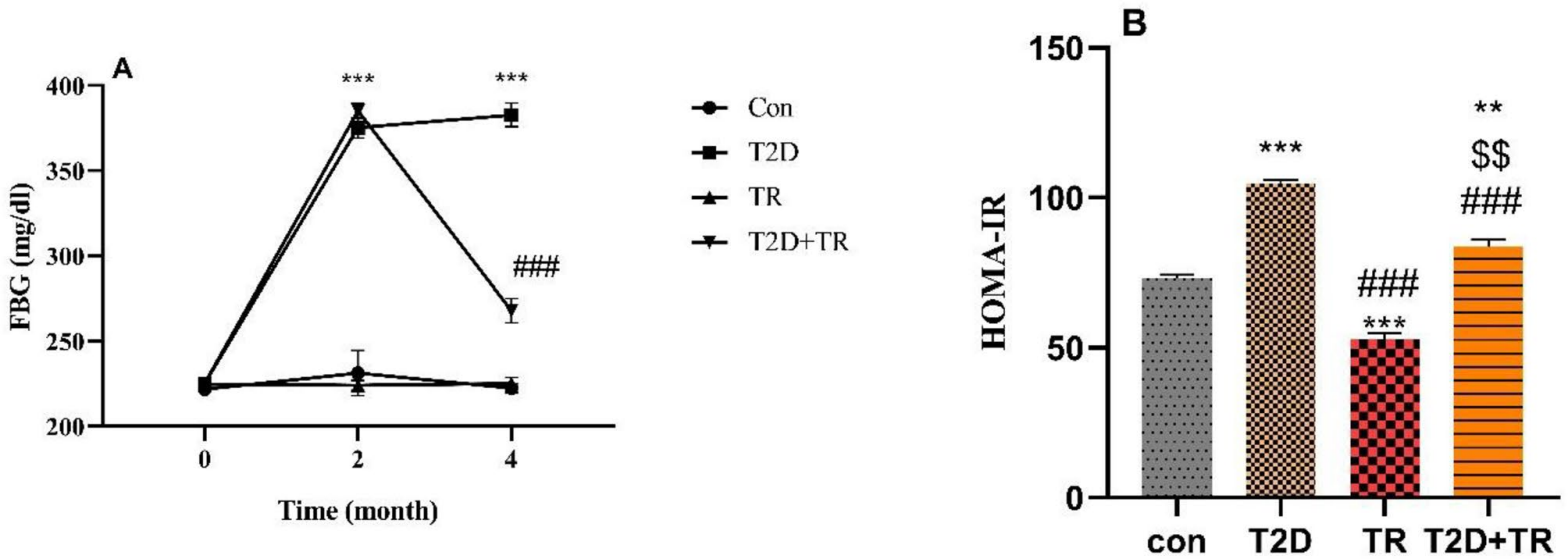
The effects of T2D and HIIT on fasting blood glucose (**A**) before starting the intervention (month 0), after diabetes induction (2 months of high-fat diet and STZ injection) (month 2), and 48 h after the last training session (month 4) in experimental groups and HOMA-IR (**B**) (mean ± SD). (n = 7 in each group). FBG: Fasting blood glucose, Con: control, T2D: Type 2 diabetic (STZ injected), TR: training only, and T2D + TR: Type 2 diabetic + Training. ***P < 0.001 & **P < 0.01 compared to the Con group. ###*P* < 0.001 compared to the T2D group. $$*P* < 0.01 compared to the TR group.

Our results showed that FBG was significantly increased in diabetic rats compared to baseline (month 0) in the T2D and T2D + TR groups (*P* < 0.001), with no notable differences between these groups. In addition, HIIT significantly reduced blood glucose levels (*P* < 0.001) (Fig. 1A). The homeostasis model assessment-estimated insulin resistance (HOMA-IR) has been widely used for the estimation of insulin resistance in researches. Compared with the gold standard euglycemic clamp method for quantifying insulin resistance, quantification using HOMA-IR is more convenient. HOMA-IR, an index of IR, was higher in T2D and T2D + TR, but lower in the TR group than in the Con group. In addition, HOMA-IR was higher in the T2D group than in the TR and T2D + TR groups (*P* < 0.001). But it was higher in the T2D + TR group than in the TR group (P < 0.01) (Fig. 1B).

### Blood pressure and cardiac function

**Figure 2:**
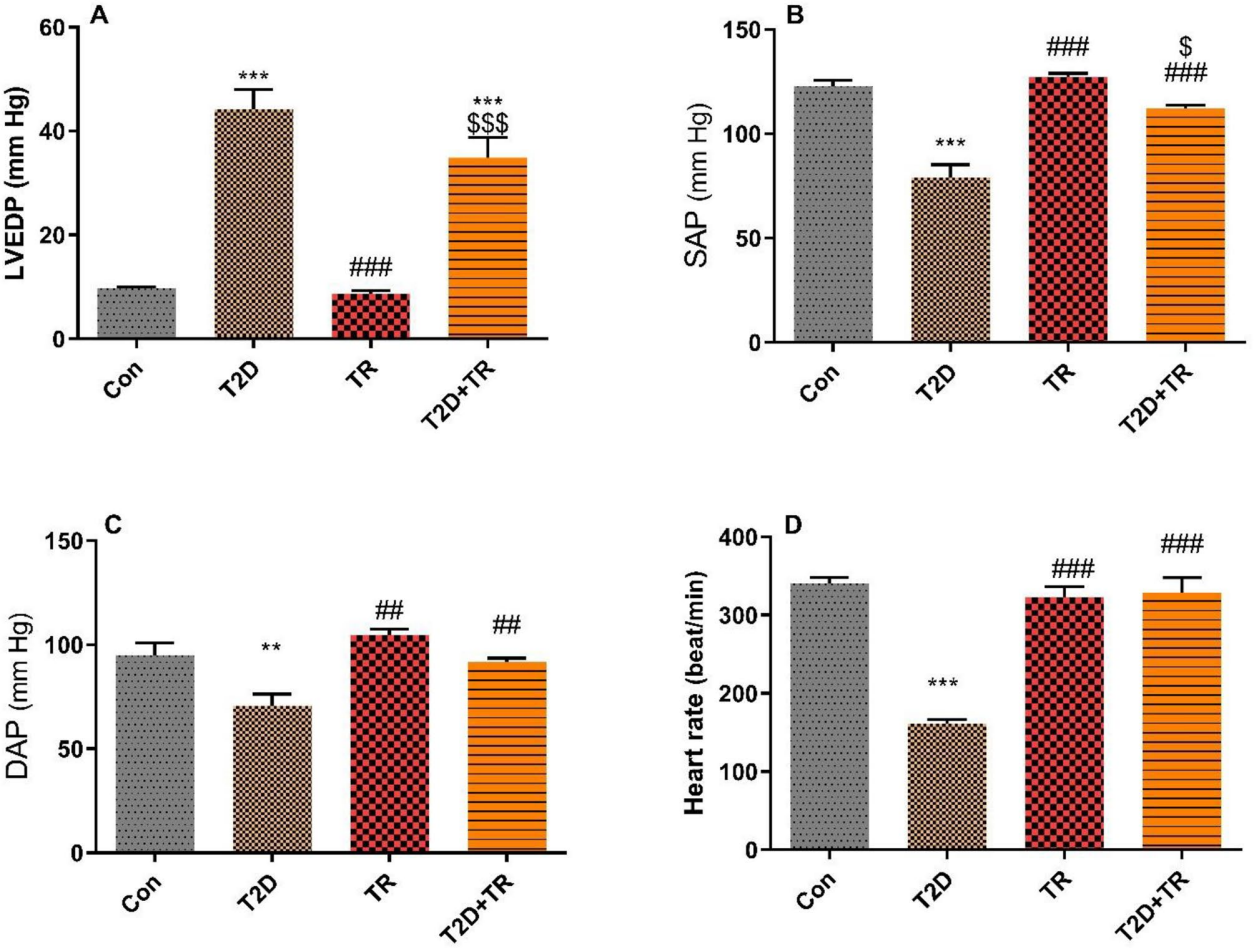
The effects of T2D and HIIT on Left ventricular end-diastolic pressure (LVEDP) (**A**), systolic (**B**), diastolic (**C**) pressures, and Heart rate (**D**) in experimental groups (n = 7 in each group). Con: control, T2D: Type 2 diabetic, TR: Training, and T2D + Ex: Type 2 diabetic + Training.**P < 0.01 & ***P < 0.001 compared to the Con group. ###*P* < 0.001 & ##*P* < 0.01 compared to the T2D group. $ *P* < 0.05 & $$$ *P* < 0.001 compared to the TR group.

**Figure 3:**
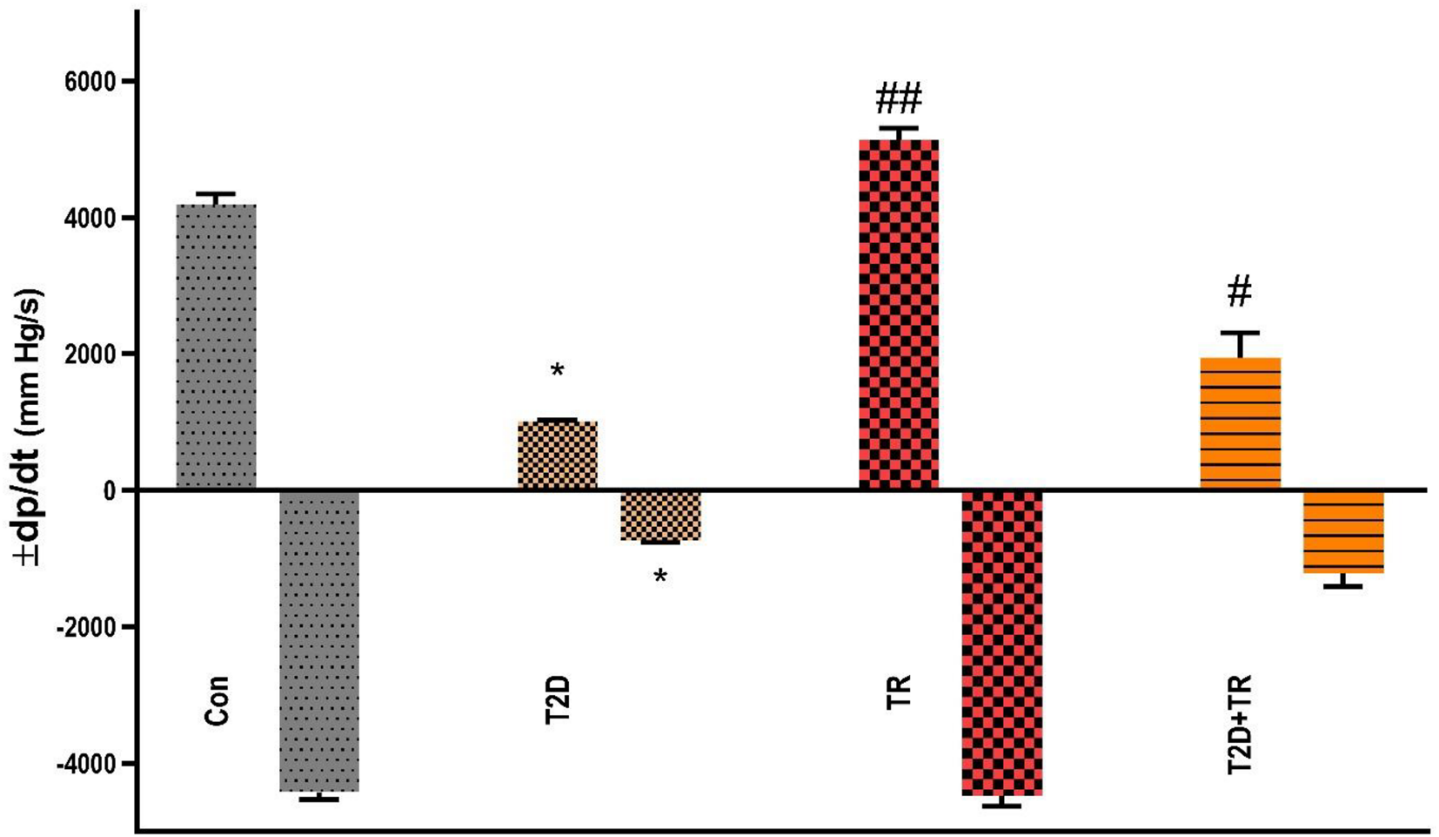
The effects of T2D and HIIT on Positive dP/dt max (+ dP/dt max) and negative dP/dt max (− dP/dt max) in experimental groups (n = 7 in each group). Con: control, T2D: Type 2 diabetic, TR: Training, and T2D + TR: Type 2 diabetic + Training. *P < 0.05 compared to the Con group. # *P* < 0.05 & ## *P* < 0.01 compared to the T2D group.

T2D decreased systolic (SAP) and diastolic arterial pressure (DAP) (*P* < 0.001 and *P* < 0.01, respectively), + dP/dt max, − dp/dt max (*P* < 0.05) and heart rate (*P* < 0.001) and increased LVEDP (P < 0.001) compared to the Con group. However, HIIT was able to reverse these changes towards normal values (Figs. 2 and 3). SAP, DAP and heart rate were lower in T2D compared to TR and T2D + TR (*P* < 0.01). DAP and heart rate showed no significant difference between TR and T2D + TR, but SAP was higher in TR compared to T2D + TR (*P* < 0.05). Maximum and minimum dp/dt (± dp/dt as an index of cardiac contractility) were reduced in the T2D group compared to the control group, while HIIT increased ± dp/dt.

### Molecular changes

**Figure 4:**
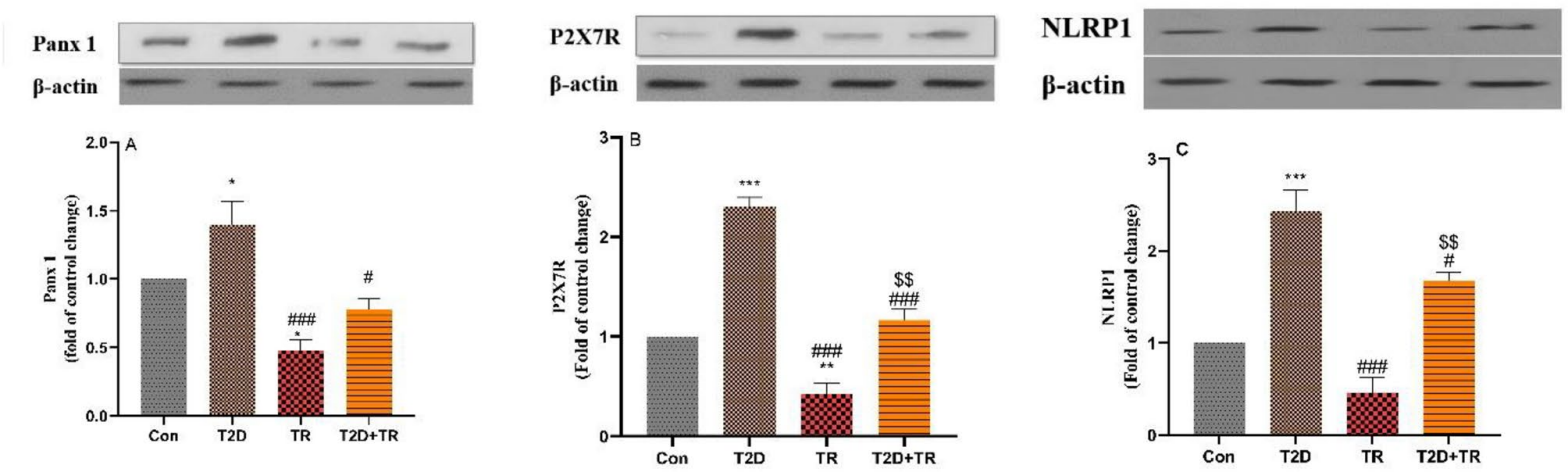
The effects of T2D and HIIT on Heart protein levels of Panx 1 (**A**), P2X7R (**B**), and NLRP1 (**C**) (mean ± SD) in experimental groups (n = 7 in each group). The blot image in each figure is a representative of seven protein extracts. Panx 1: pannexin 1, Con: control, T2D: Type 2 diabetic, TR: Training, and T2D + TR: Type 2 diabetic + Training. *P < 0.05 & ***P < 0.001 & **P < 0.01 compared to the Con group. # *P* < 0.05 & ### *P* < 0.001 compared to the T2D group. $$ *P* < 0.01 compared to the TR group.

**Figure 5:**
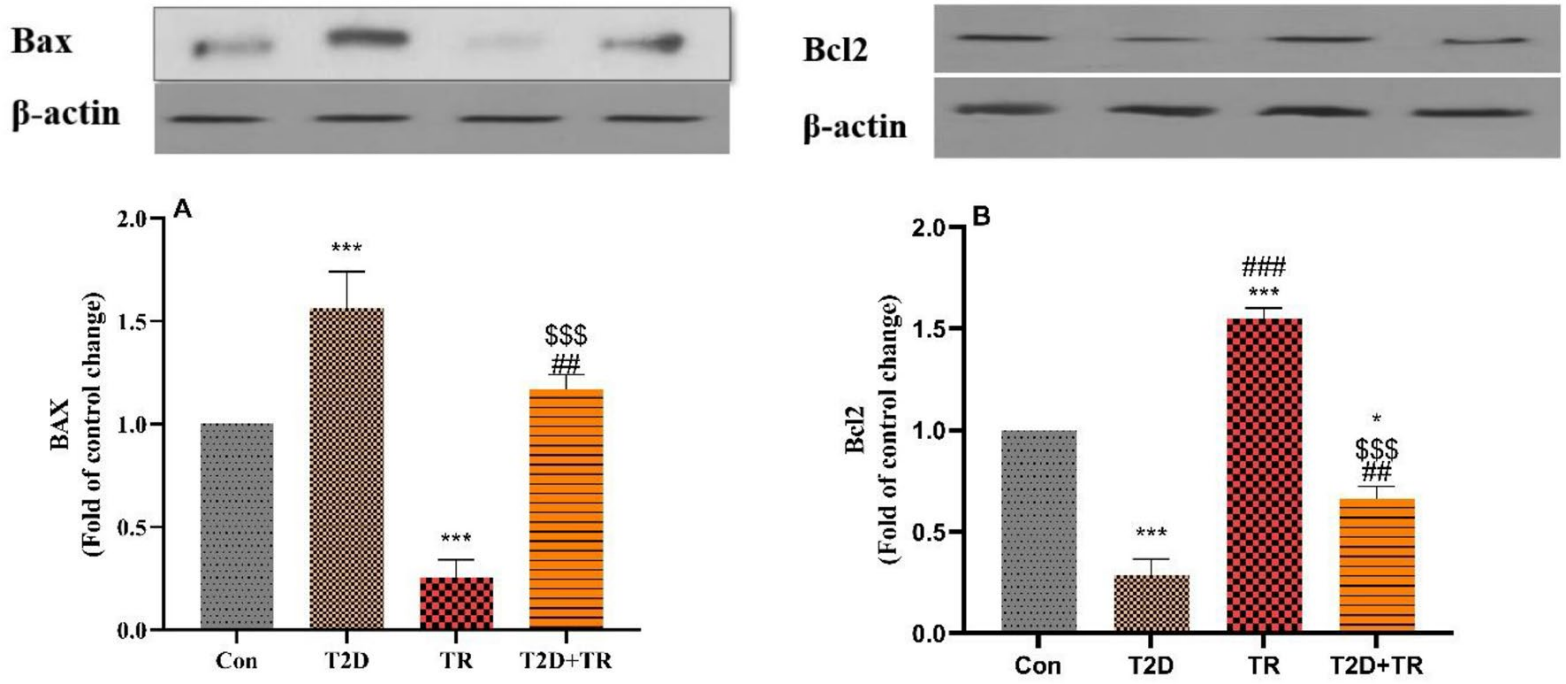
The effects of T2D and HIIT on Heart protein levels of BAX (**A**) and Bcl2 (**B**) (mean ± SD) in experimental groups (n = 7 in each group). The blot image in each figure is a representative of seven protein extracts. Con: control, T2D: Type 2 diabetic, TR: Training, and T2D + TR: Type 2 diabetic + Training. ***P < 0.001 & *P < 0.05 compared to the Con group. ## *P* < 0.01 & ### *P* < 0.001 compared to the T2D group. $$$ *P* < 0.001 compared to the TR group.

The expression of P2X7R, NLRP1 and Panx1 differed significantly between the groups, with higher expression in the T2D group compared to the Con group (*P* < 0.05 for Panx-1, *P* < 0.001 for P2X7R and NLRP-1). In addition, P2X7R and Panx1 had lower values in the TR group compared to the Con group (*P* < 0.05 for Panx-1, *P* < 0.01 for P2X7R). The expression of these parameters was significantly lower in T2D + TR compared to the T2D group (*P* < 0.05 for Panx-1 and NLRP-1 and *P* < 0.001 for P2X7R) (Fig. 4). Bcl2 expression was lower in the T2D group compared to the Con group, while BAX expression was higher in the T2D group compared to the Con group (*P* < 0.001). In the TR group, the opposite trend was observed for both variables compared to the Con group. The Bcl2 level was higher in the T2D + TR group than in the T2D group, while the BAX level was lower in the T2D + TR group than in the T2D group (*P* < 0.01) (Fig. 5). Panx1, P2X7R, NLRP-1 and BAX levels were also higher in the T2D + TR group, while Bcl2 levels were significantly lower in the TR group.

**Figure 6:**
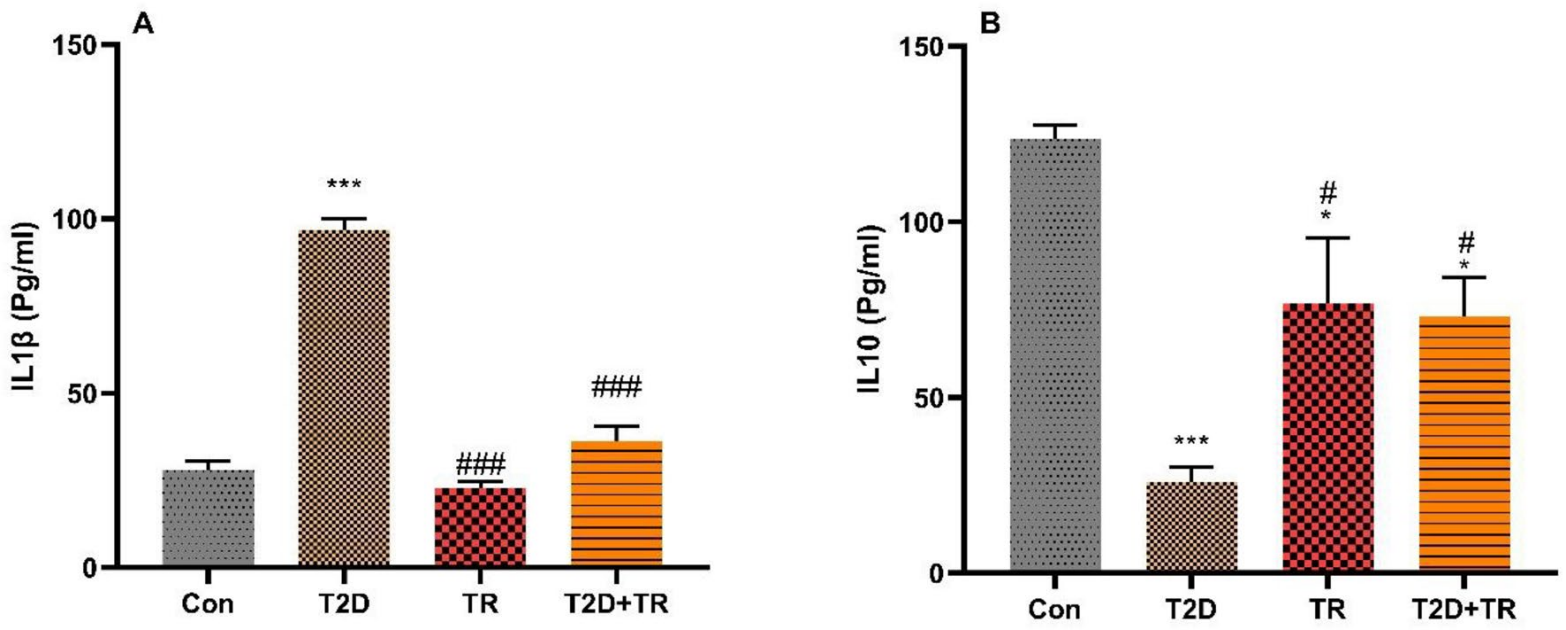
The effects of T2D and HIIT on Heart levels of IL1β (A) and IL-10 (B) (mean ± SD) in experimental groups (n = 7 in each group). Con: control, T2D: Type 2 diabetic, TR: Training, and T2D + TR: Type 2 diabetic + Training. ***P < 0.001 & *P < 0.05 compared to the Con group. # *P* < 0.05 & ### *P* < 0.001 compared to the T2D group.

IL1β level was higher in the T2D group than in the other group (*P* < 0.001) (Fig. 6). In addition, IL-10 level was higher in Con compared to the other groups (*P* < 0.001 for T2D and *P* < 0.05 for TR and T2D + TR), but it was lower in T2D compared to T2D + TR and TR (*P* < 0.05) (Fig. 6).

### Histopathological findings

**Figure 7:**
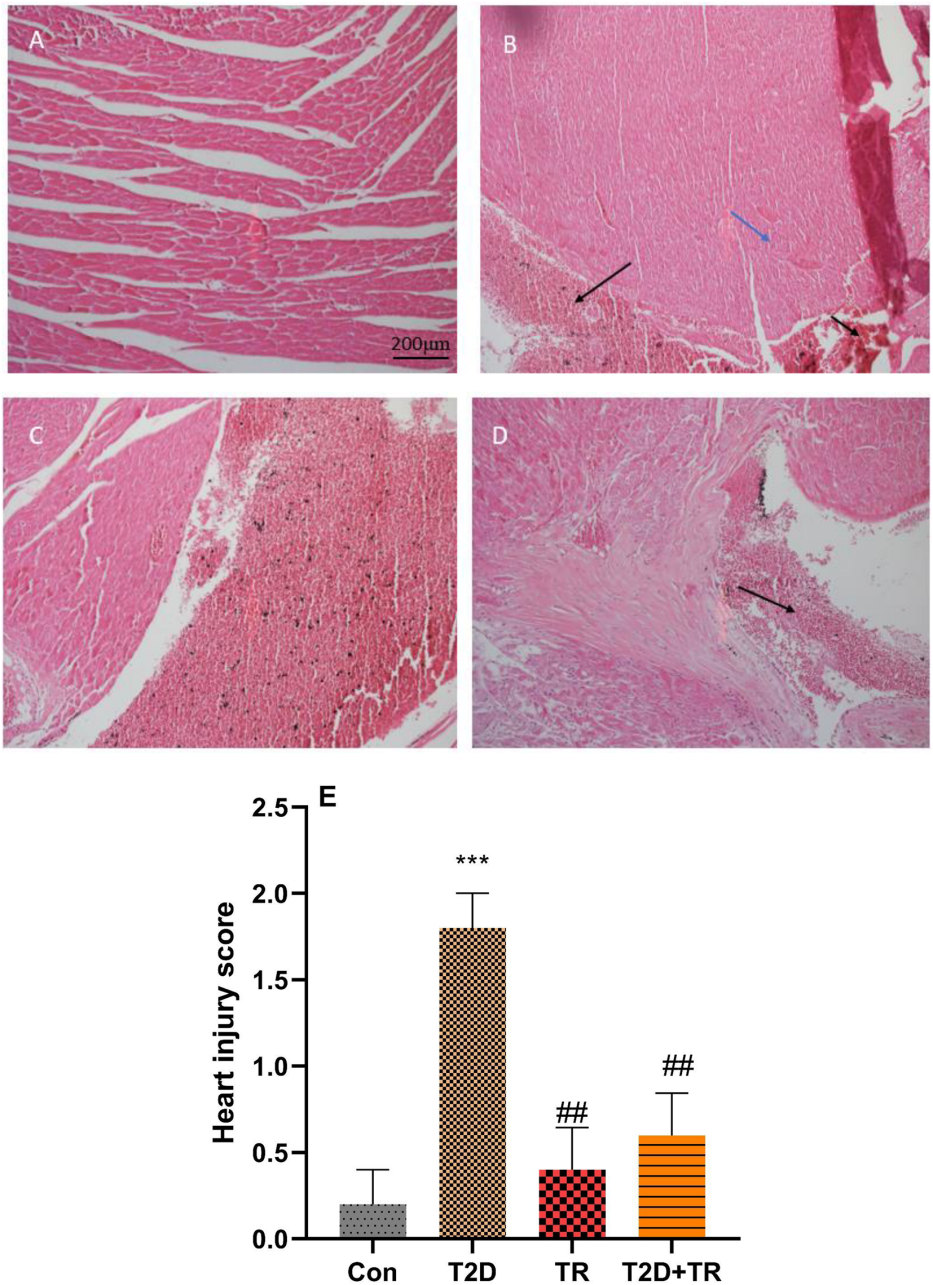
The effects of T2D and HIIT on heart injury. Micrographs of the heart stained with H&E. Data are presented as mean ± SD. Con: A: control; B: T2D C: TR; D: T2D + TR. Co: control, T2D: Type 2 diabetic, TR: Training, and T2D + TR: Type 2 diabetic + Training. Black arrows indicate congestion and blue arrows indicate hypertrophy. ***P < 0.001 compared to the Con group. ## P < 0.01 compared to the T2D group.

**Figure 8:**
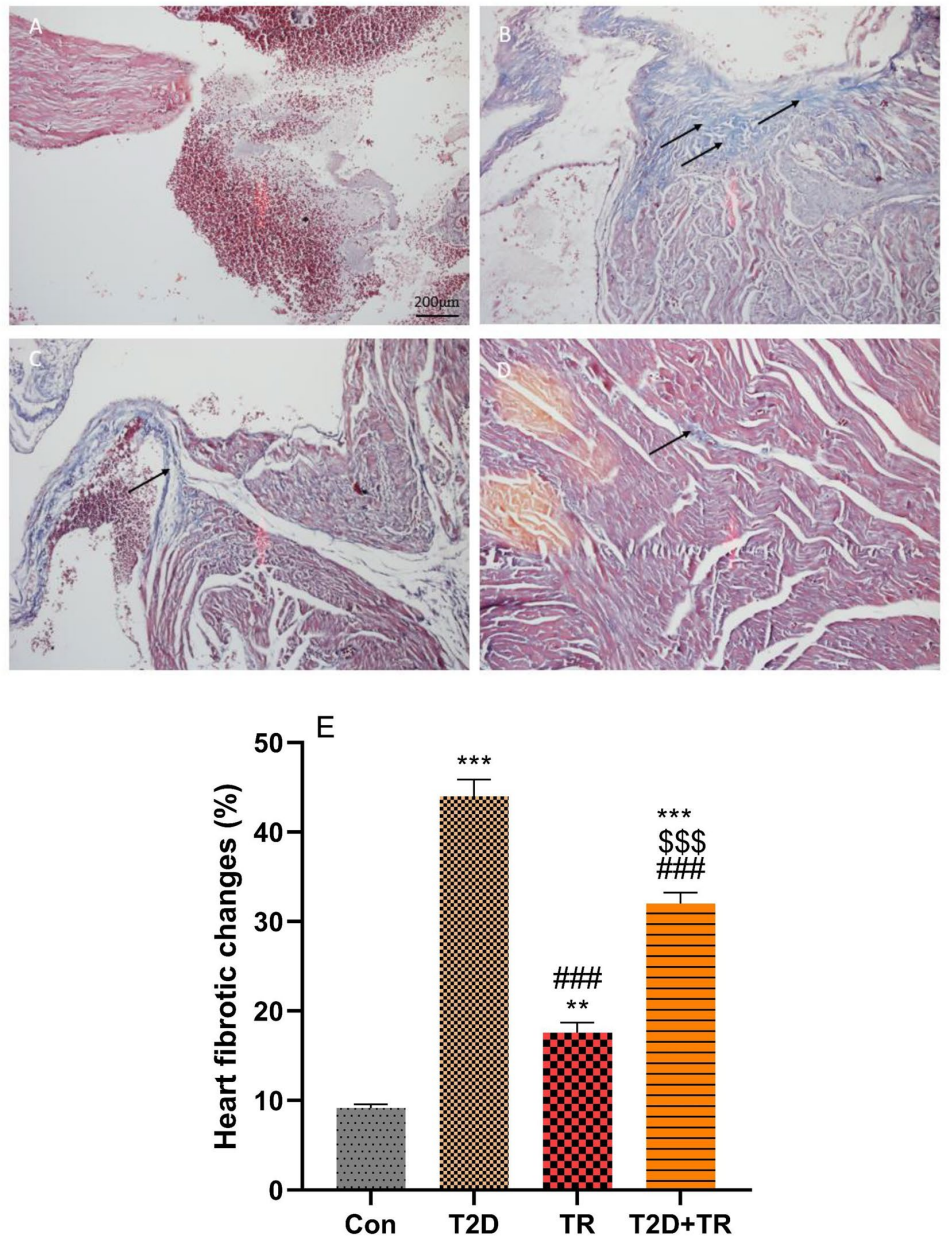
The effects of T2D and HIIT on heart fibrosis. Micrographs of the heart stained with Masson trichrome showed heart fibrosis. Data are presented as mean ± SD. Con: A: control; B: T2D C: TR; D: T2D + TR. Co: control, T2D: Type 2 diabetic, TR: Training, and T2D + TR: Type 2 diabetic + Training. Black arrows indicate fibrotic changes. ***P < 0.001 & **P < 0.01 compared to the Con group. ### *P* < 0.001 compared to the T2D group. $$$ *P* < 0.001 compared to the TR group.

Our results showed that the cardiac lesion score was higher in T2D than in the other groups (*P* < 0.001 for Con and *P* < 0.01 for TR and T2D + TR). In addition, the percentage of fibrosis was higher in T2D than in the other groups (*P* < 0.001). This percentage was also higher in the TR and T2D + TR groups compared to Con (*P* < 0.01 and *P* < 0.001, respectively) and in T2D + TR compared to TR (*P* < 0.001) (Figs. 7 and 8).

## Discussion

The aim of this study was to investigate the effect of HIIT on molecular, functional and histopathological changes in the hearts of rats with T2D, with a particular focus on ATP-releasing channels. Our results showed that T2D led to a significant decrease in systolic and diastolic arterial pressure, heart rate, ± dP/dt max and an increase in LVEDP. In addition, T2D induced cardiac inflammation and apoptosis in diabetic rats, as well as increased expression of NLRP1 (an inflammasome), Panx-1 and P2X7R. In addition, T2D led to an increased heart lesion score and increased fibrosis in cardiac tissue. However, HIIT appeared to attenuate the negative effects of diabetes on cardiac function, histopathology, NLRP1, ATP-releasing channels, inflammation and apoptosis.

The decrease in + dp/dt max and -dp/dt max, indicating decreased cardiac contractility and relaxation velocity, associated with an increase in LVEDP in the T2D group, is consistent with previous studies^53,54^. In addition, our results showed that HIIT could restore cardiac function to near-normal levels. Previous studies^53,54^ have shown that various exercise protocols, including endurance training and low-intensity training, can ameliorate cardiac impairments in diabetic rats. Furthermore, training has been associated with delaying or preventing the progression of cardiac impairments^55,56^. Our results are consistent with these findings and suggest that training, including HIIT, may have a beneficial effect on cardiac function in T2D.

The presence of fibrosis in our study is consistent with previous studies^57–59^, in which inflammation has been proposed as one of the mechanisms contributing to fibrosis after diabetes^60^. Inflammatory processes have been linked to cardiac fibrosis^61^, which is confirmed by our results.

T2D and HIIT appeared to have opposing effects on Panx-1 expression in our study. Increased Panx-1 expression may trigger a signaling cascade leading to an increase in inflammatory factors such as IL-1β^62^. Kienitz et al.^63^ showed that IL-1β levels in the heart are low under normal conditions, but its expression increases significantly under inflammatory conditions. In addition, Panx-1 channels are functionally linked to P2X7 receptors, and increased Panx-1 activity can enhance the effect of ATP on these receptors^64^. There is also evidence of a link between Panx1 and NLRP1 expression^65^. Rami et al.^66^ demonstrated that endurance training could effectively inhibit the overexpression of Panx-1 and NLRP-1 in the hippocampus of diabetic rats, which is consistent with our results.

P2X7R, a key modulator of IL-1β, has been implicated in diabetic nephropathy^67^ and has significant effects on the cardiovascular system11. Our results suggest that T2D increases the expression of P2X7R, which is consistent with previous studies. Training, including HIIT, has been shown to attenuate the expression of P2X7R and improve cardiac remodeling in diabetic animals^32,68^, which is consistent with our findings.

Our results showed that HIIT decreased NLRP1 expression in the heart of diabetic rats. Similarly, Kazemi et al.^69^ reported that aerobic exercise attenuated the adverse effects of deep-heated oils on the expression of NLRP3, thereby improving myocardial degradation and ameliorating structural changes in myocytes. Furthermore, Sun and Ding^70^ proposed that diabetic cardiomyopathy is an inflammatory disease exacerbated by NLRP3 inflammasome-mediated release of IL-1β and IL-18. They proposed that a chronic training intervention is an effective preventive and curative approach to ameliorate diabetic cardiomyopathy by modulating the NLRP3 inflammasome and subsequently reducing inflammation, a finding confirmed by our results (i.e., decreased IL1β and increased IL10 levels). Increased IL1β levels^71,72^ and decreased IL10 levels^73,74^ in the hearts of animals with T2D have been well documented in other studies.

Inflammatory cytokines have been hypothesised to induce apoptosis^75^. Apoptosis can also be triggered by the binding of ligands (such as inflammatory cytokines) to specific death receptors on the cell membrane, leading to the recruitment of adaptor proteins and activation of cysteine protease cascades that ultimately induce cell death^76^. Compared to non-diabetics, diabetics exhibit an 85-fold, 61-fold and 26-fold increase in apoptosis of cardiomyocytes, endothelial cells and fibroblasts, respectively^77^. Animal studies have shown that hyperglycemia promotes cardiomyocyte apoptosis, which is associated with increased collagen deposition and impaired systolic and diastolic function^78,79^. Many studies have shown that Bax, a proapoptotic factor, is upregulated in the heart after diabetes, whereas Bcl2, an antiapoptotic factor, is downregulated^59,80^. Our results also showed increased levels of Bax and decreased levels of Bcl2 in diabetic rats.

In agreement with our findings, numerous studies have shown that physical training can modulate apoptosis factors^23,81–83^. Eight weeks^84^ and four weeks^85^ of HIIT have been shown to increase the expression of the Bcl2 gene in the hearts of diabetic rats. As previously mentioned, training can modulate inflammation^86^, and it appears that the primary protective effect of training against apoptosis may be via modulation of inflammation. One study proposed that reducing ROS may also help to reduce apoptotic factors by preventing the release of mitochondrial cytochrome C^87^. Apoptosis is thought to contribute to cardiac dysfunction^76^.

## Conclusion

**Figure 9:**
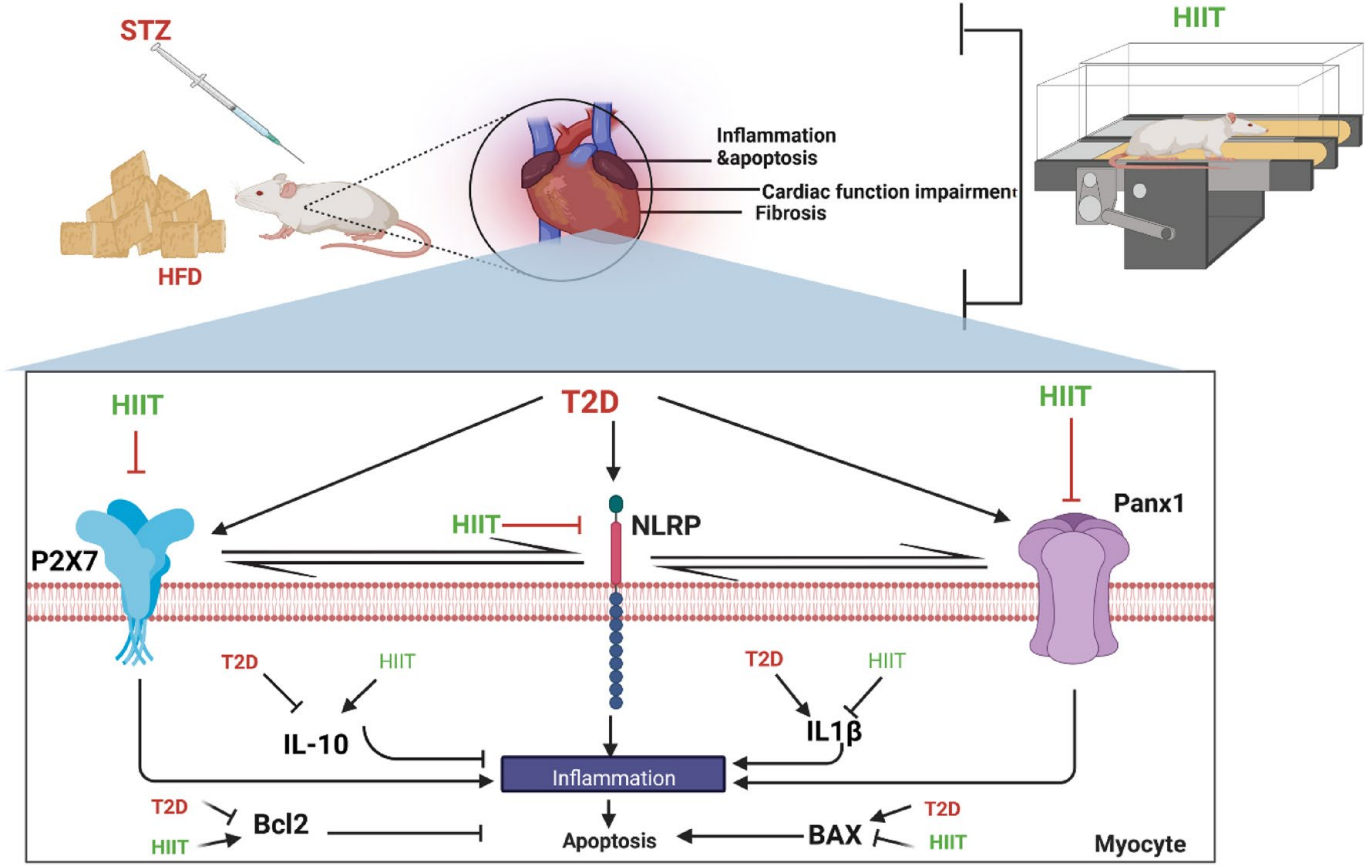
HIIT could improve diabetic hearts through modulation of ATP-releasing channel expression, inflammation, and apoptosis.

Our study suggests that HIIT has anti-inflammatory and anti-apoptotic effects on the heart of T2D rats. These effects are possibly mediated by the modulation of ATP-dependent channels such as Panax 1 and P2X7R as well as NLRP1 as inflammasome receptors. These molecular findings were supported by functional (improvement in blood pressure and heart rate) and histopathologic (improvement in cardiac injury and fibrosis) results (Fig. 9).

### Study limitations and suggestions for future studies

Some important limitations of our study should be noted. First, we did not measure visceral fat mass, which could potentially mediate the anti-inflammatory effects of exercise. It is well known that visceral fat is metabolically active and is associated with increased inflammation. Incorporating measurements of visceral fat mass into future studies could provide a more comprehensive understanding of the effects of training on inflammation in the context of diabetes.

Second, we did not measure cardiac mass index and intra/extracellular ATP content, which could have provided valuable insights into the underlying mechanisms of the observed effects. The cardiac mass index could help assess potential structural changes in the heart, while measurements of ATP content could shed light on the role of ATP-dependent channels in the observed molecular changes. Incorporating these measurements into future research could improve our understanding of the mechanisms by which exercise influences cardiac function in diabetes.

Third, studying the indicators of cardiac function of animals using echocardiography as a non-invasive and indirect method could have provided us with clear information about the structure and function of the heart, and the lack of echocardiography is one of the limitations of our work and can be used for future studies.

Fourth, it is suggested to use transmission electron microscopy in future studies to investigate structural details related to cardiac tissue.

Fifth, it is suggested that future studies measure the levels of myocardial injury enzymes.

## Ethics approval

All stages of keeping and sacrificing the animals were performed according to the rules of the ethics committee of Kerman University of Medical Sciences (Ethic code: IR.KMU.AH.REC.1400.081).

### Supplementary Information

Supplementary Information.

## Data Availability

The original contributions presented in the study are included in the article/supplementary material, and further inquiries can be directed to the corresponding author.
