# Supplementary material for: ATP releasing channels and the ameliorative effects of high intensity interval training on diabetic heart: a multifaceted analysis
Source: Sci Rep. 2024 Mar 26;14:7113. doi: 10.1038/s41598-024-57818-0 (PMC10965991; doi:10.1038/s41598-024-57818-0)

BAX (7 samples , Triplicated)

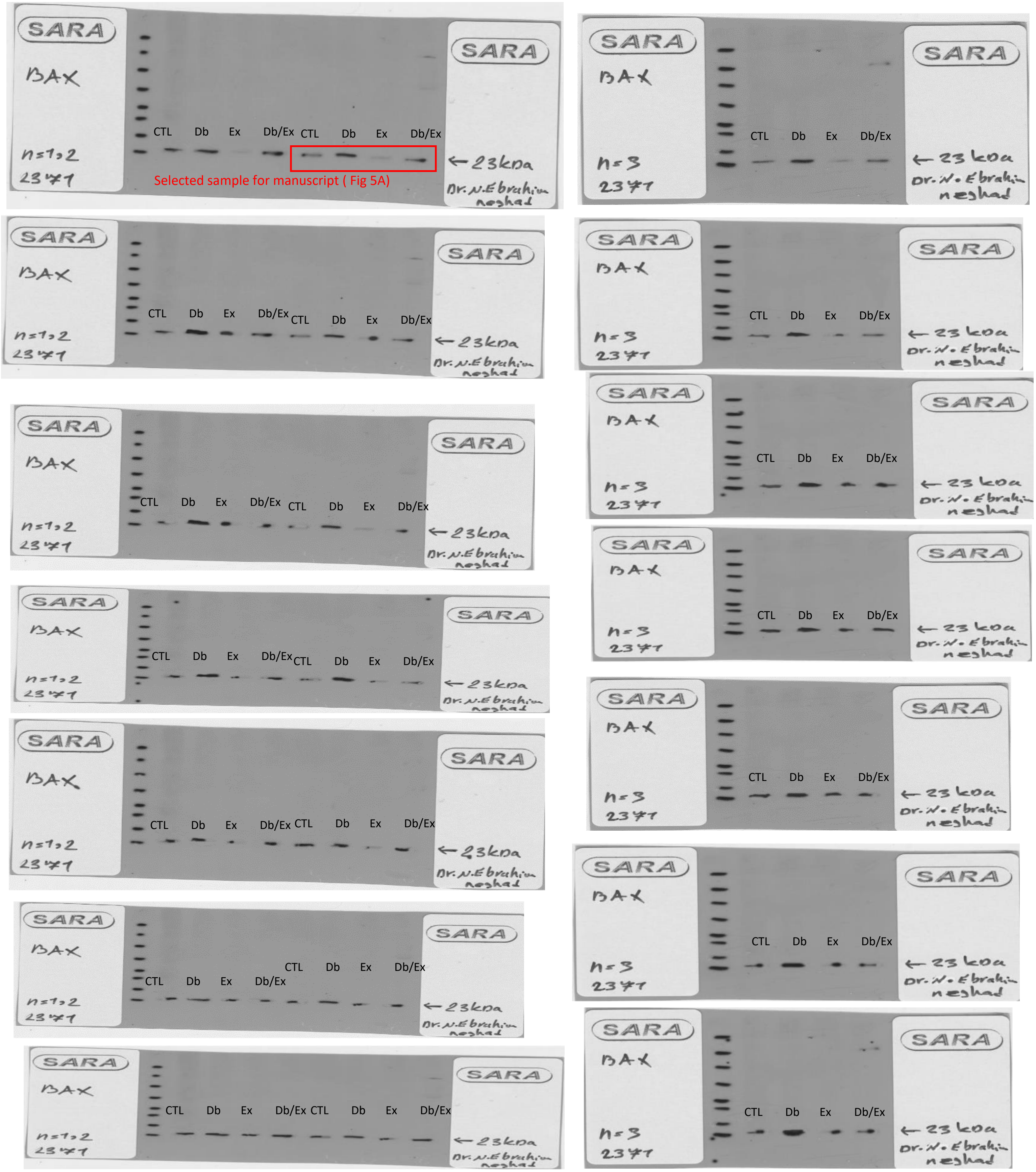

Bcl2(7 samples , Triplicated)

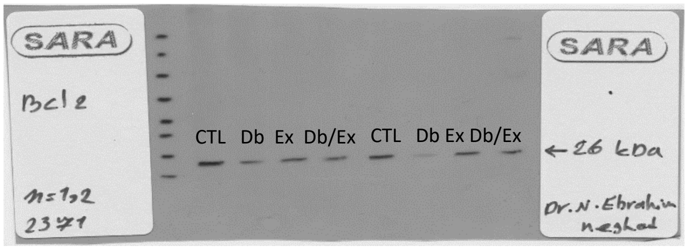

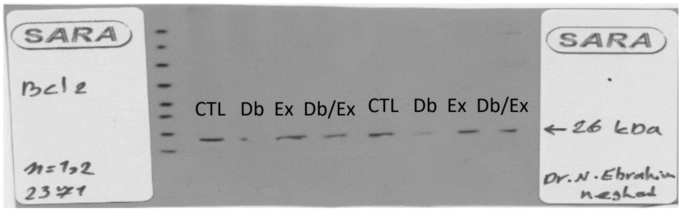

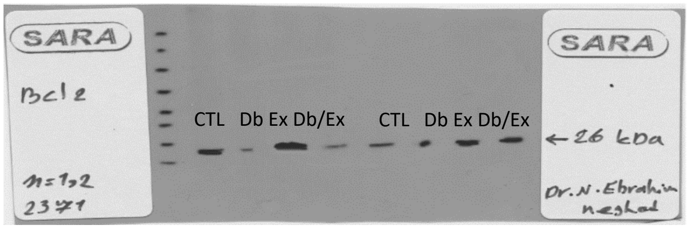

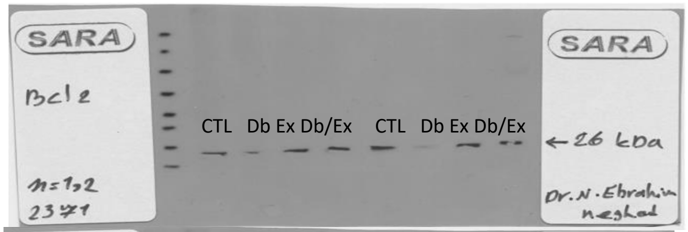

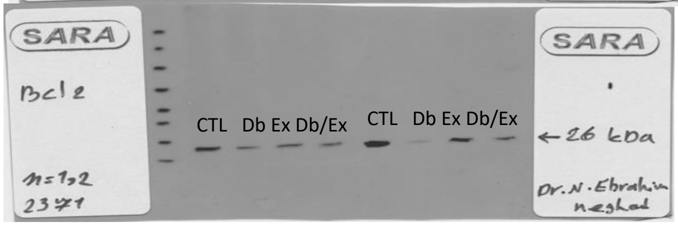

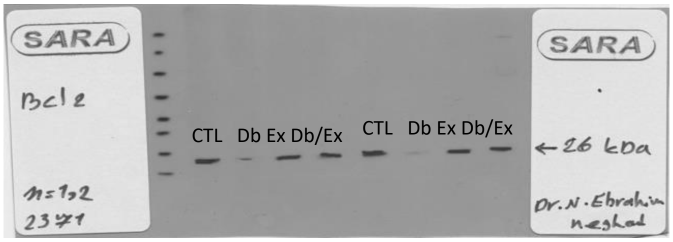

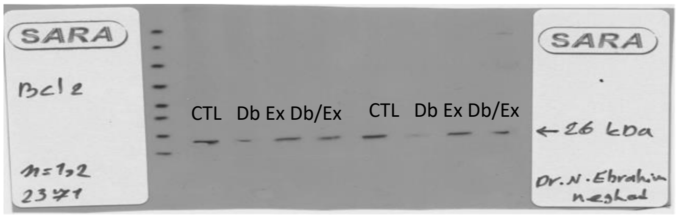

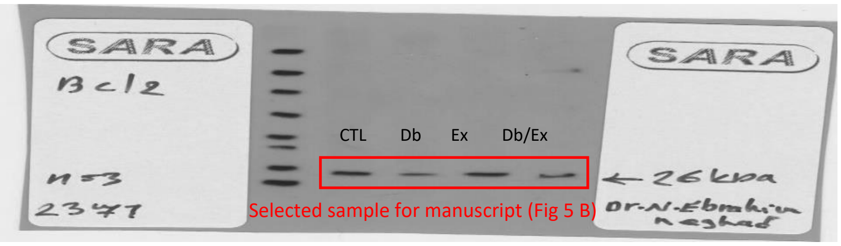

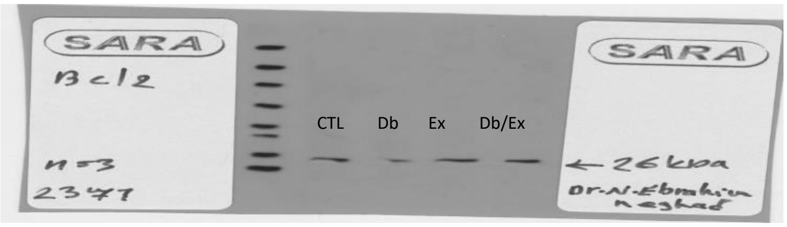

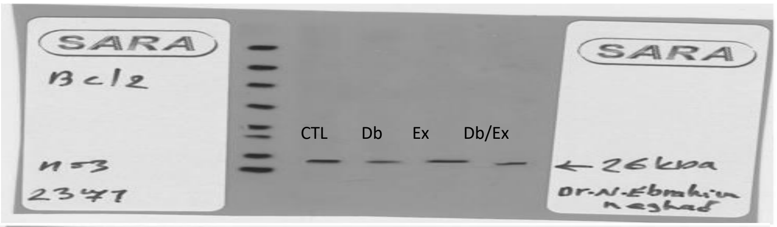

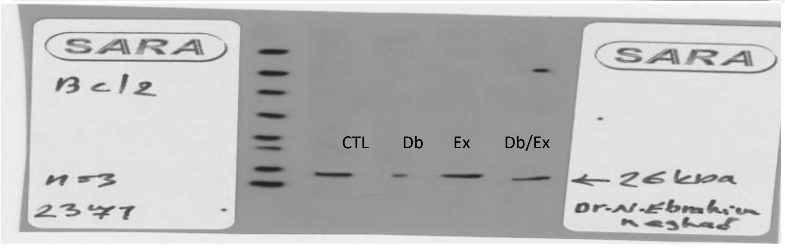

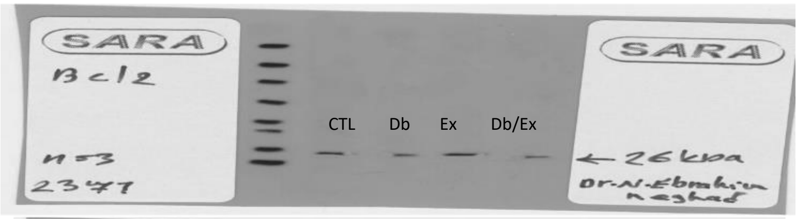

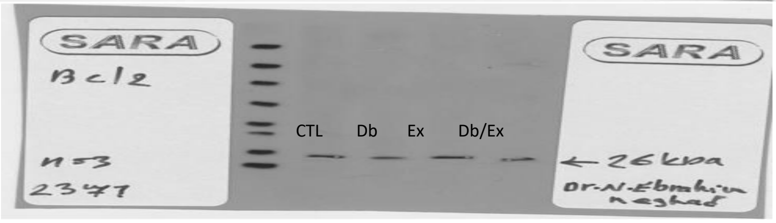

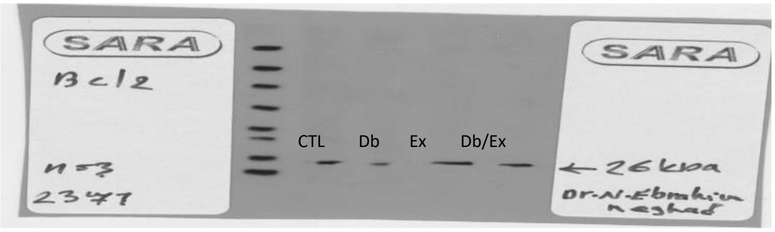

P2X7R (7 samples , Triplicated)

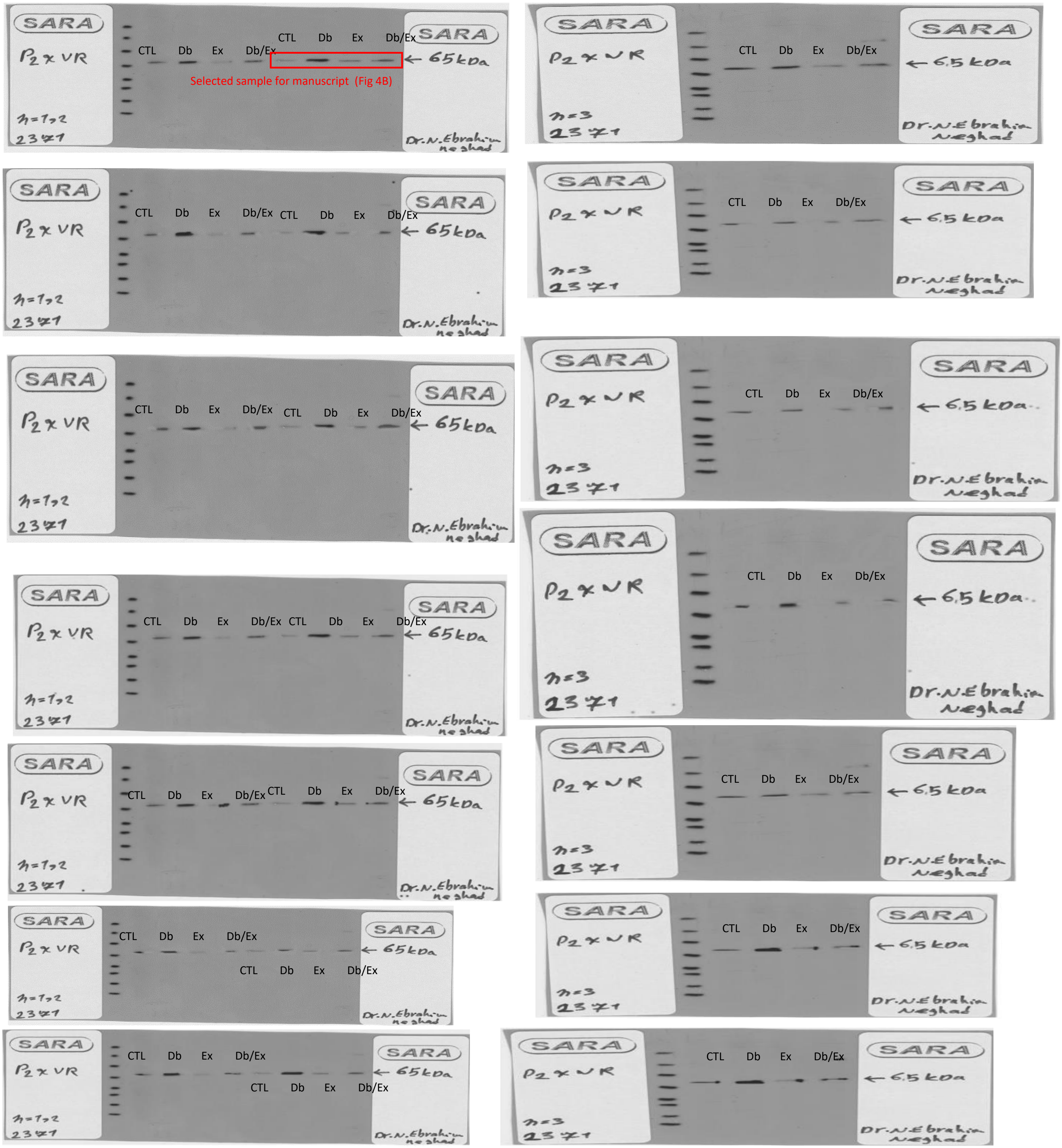

NLRP1 (7 samples, Triplicated)

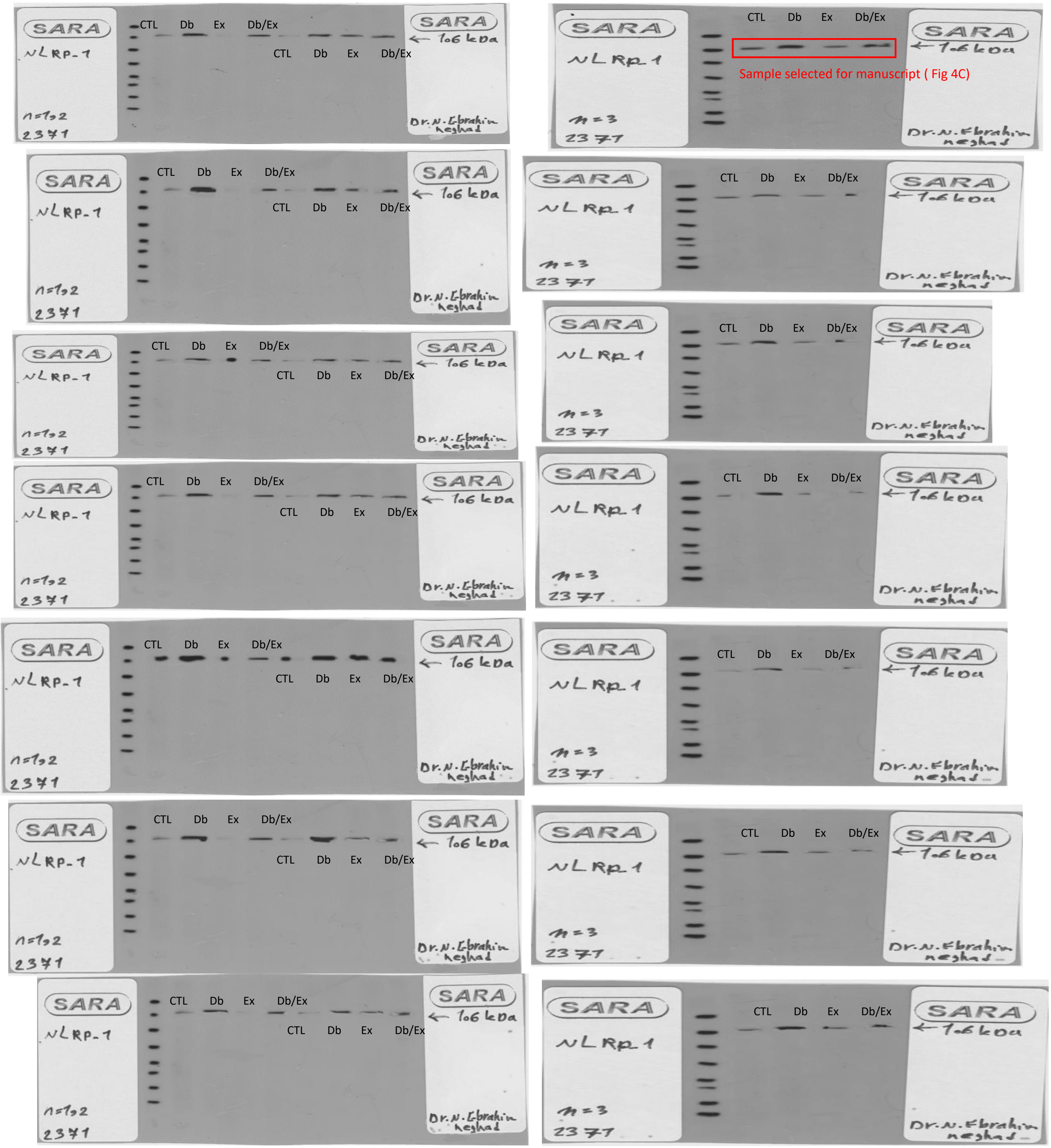

Pannexin1 (7 samples, Triplicated)

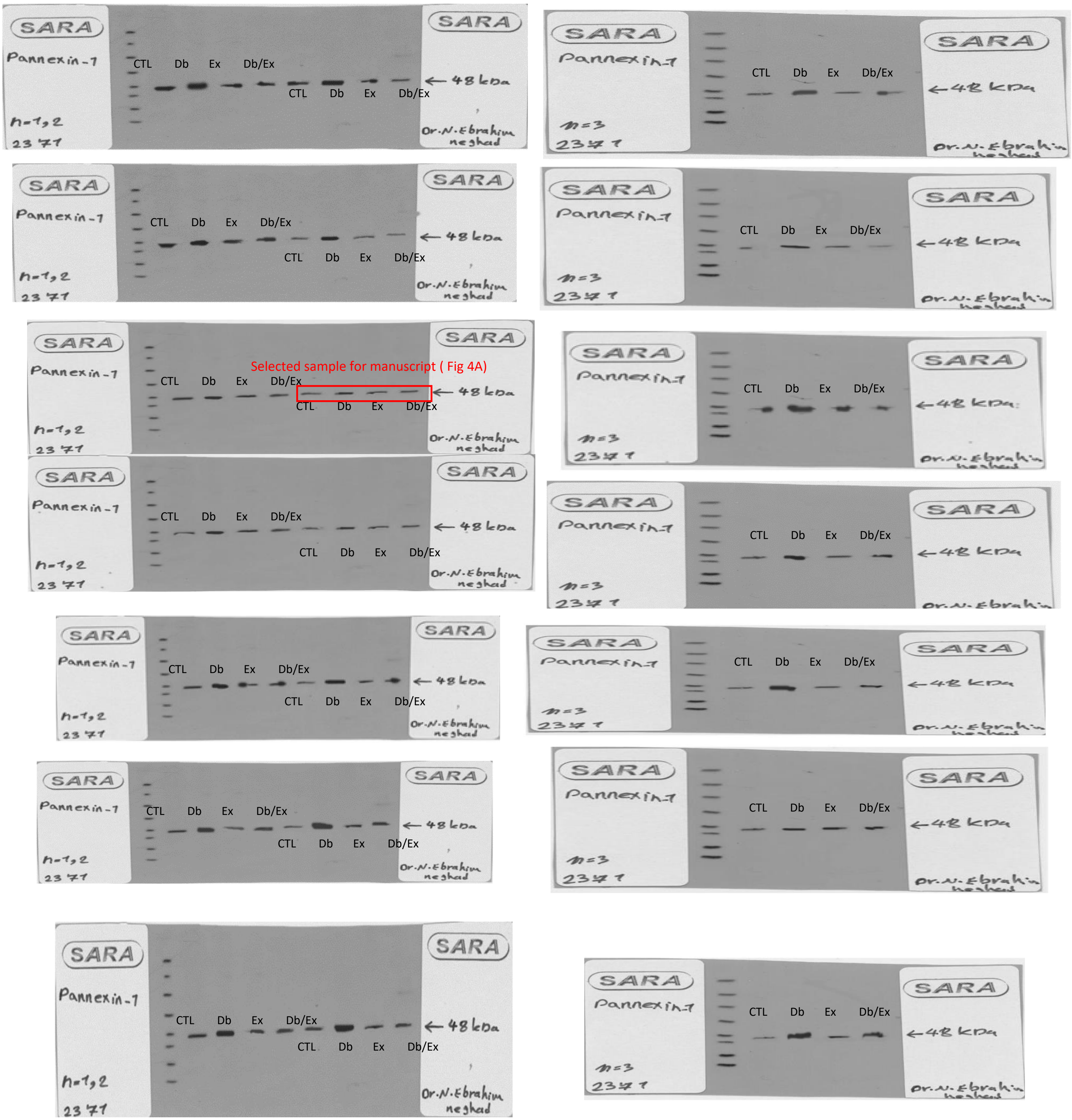

B-Actin (7 samples , Triplicated)

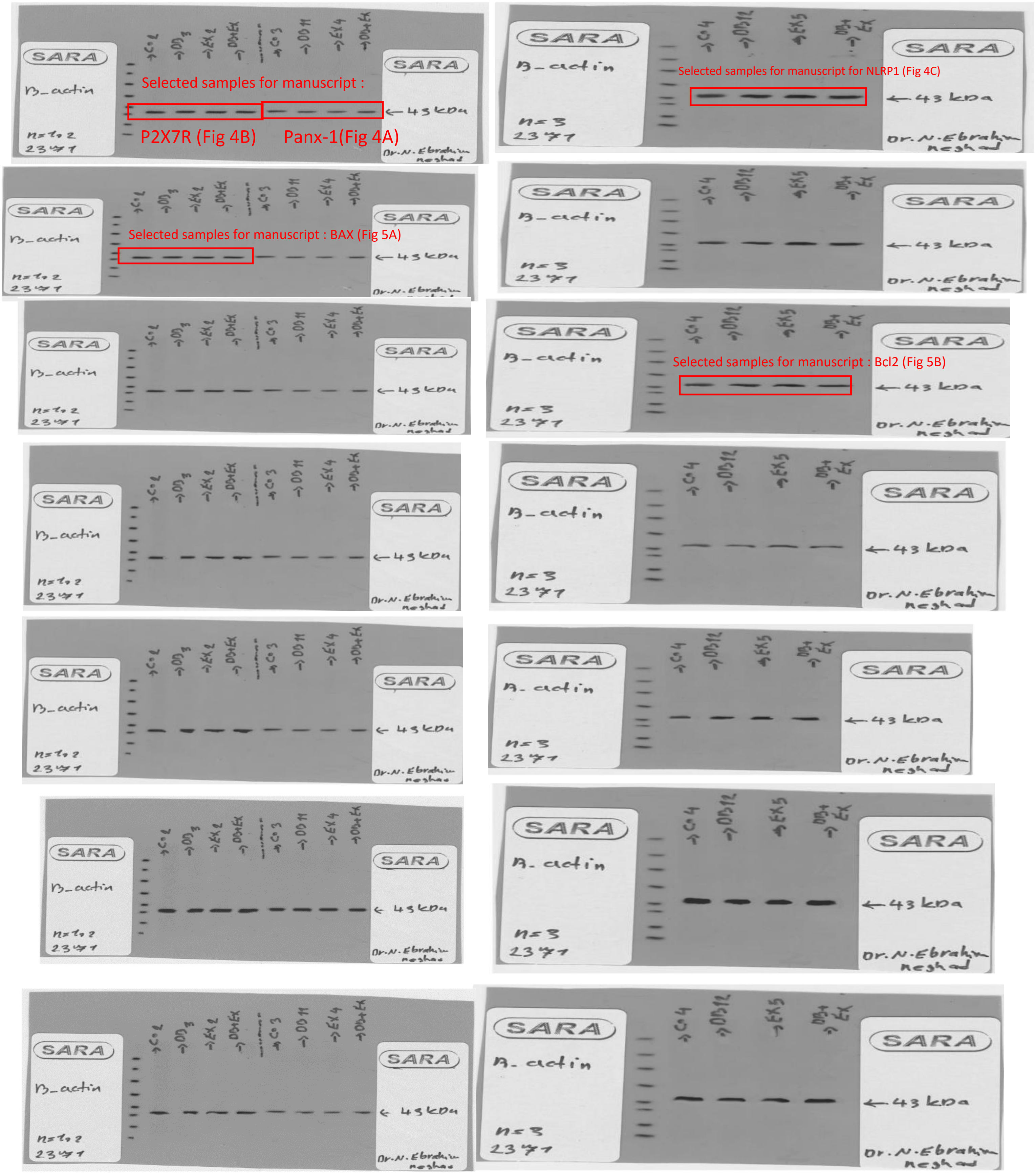

Supplement: Supplementary file 1 — Supplementary Information. [file 41598_2024_57818_MOESM1_ESM.pdf]
